# Adult Wilms' Tumor: Case Report and Literature Review

**DOI:** 10.7759/cureus.15524

**Published:** 2021-06-08

**Authors:** Camilo Vallejo Yepes, Marcela Bermudez, Diego Camacho-Nieto, Jorge Mesa, Ricardo Bruges

**Affiliations:** 1 Oncology, Instituto Nacional De Cancerología, Bogota, COL; 2 Oncology, Instituto Nacional de Cancerología, Bogotá, COL; 3 Urology, Hospital Universitario Mayor - Méderi/Universidad Del Rosario, Bogotá, COL; 4 Pathology, Instituto Nacional de Cancerología, Bogotá, COL

**Keywords:** wilms tumor, adult, nephroblastoma, case report, neoplasms

## Abstract

Wilms' tumor is childhood’s most common renal tumor, and its presentation in the adult age is extremely rare. Due to the low frequency in adults, no standard management guidelines are available for this population, also the natural history of the disease and management is unclear. We present a case report of a 31-year-old woman with metastatic Wilms' tumor, with lymph node, lung and liver involvement; systemic treatment with chemotherapy was started, with complete clinical response. Finally, a literature review is performed to showcase the differences in the clinical course, prognosis, and treatment alternatives, in adult disease compared to childhood.

## Introduction

Wilms' tumor, also known as nephroblastoma, is the main cause of solid abdominal neoplasms in children younger than six years, with a reported annual incidence of 8-10 cases per million persons; hence, this tumor shows a clear predominance for pediatric age, with 90% of cases being diagnosed in patients younger than five years [1]. The occurrence of this disease in the adult population is exceptional, with a reported incidence of fewer than 0.2 cases per million persons per year [1-2]. Due to its low frequency, it is rarely suspected in older patients, leading to delayed diagnosis and management, presenting with later stages at the initial diagnosis.

The standard pediatric treatment is proposed by two collaborative groups: the National Wilms Tumor Study (NWTS) and the International Society of Paediatric Oncology (SIOP). It implies a multimodal management with radical nephrectomy as the cornerstone of management, associated with exclusive chemotherapy or with concomitant radiotherapy in most patients [3]. By comparison, due to its lower incidence, no phase 3 studies or treatment guidelines are available for adult Wilms' tumor, with less than 300 cases published worldwide [4]. The scarce evidence in the literature makes the management of adult Wilms' tumor a challenge.

We present a case report of a 31-year-old woman treated in the Instituto Nacional de Cancerología in Bogotá, Colombia. The patient presented with a Wilms' tumor with metastatic disease and high tumor burden, which presented a complete clinical response to a pediatric chemotherapy scheme. Afterwards, we perform a literature review where the clinical course, prognosis, and treatment of Wilms' tumor are compared between children and adults.

## Case presentation

A 31-year-old female patient had a personal history of radical nephrectomy and adrenalectomy due to left renal mass in 2010, performed outside the Instituto Nacional de Cancerología, apparently with a complete resection and an unknown initial pathology report. The patient didn’t receive any additional management and remained under follow-up. In 2016, she consulted to the Instituto Nacional de Cancerología. In imaging studies, hepatic lesions with metastatic appearance were observed. Biopsy reported a morphology compatible with Wilms' tumor, and immunohistochemistry was positive for CK (AE1/AE3), WT1, BCL2, CD56, and negative for synaptophysin, chromogranin, CD99, S100, FLY-1, and estrogen receptor. Due to its morphology, a differential diagnosis of synovial sarcoma was proposed; hence, a fluorescence in situ hybridization (FISH) study for the SS18 gene was performed, which was negative.

**Figure 1:**
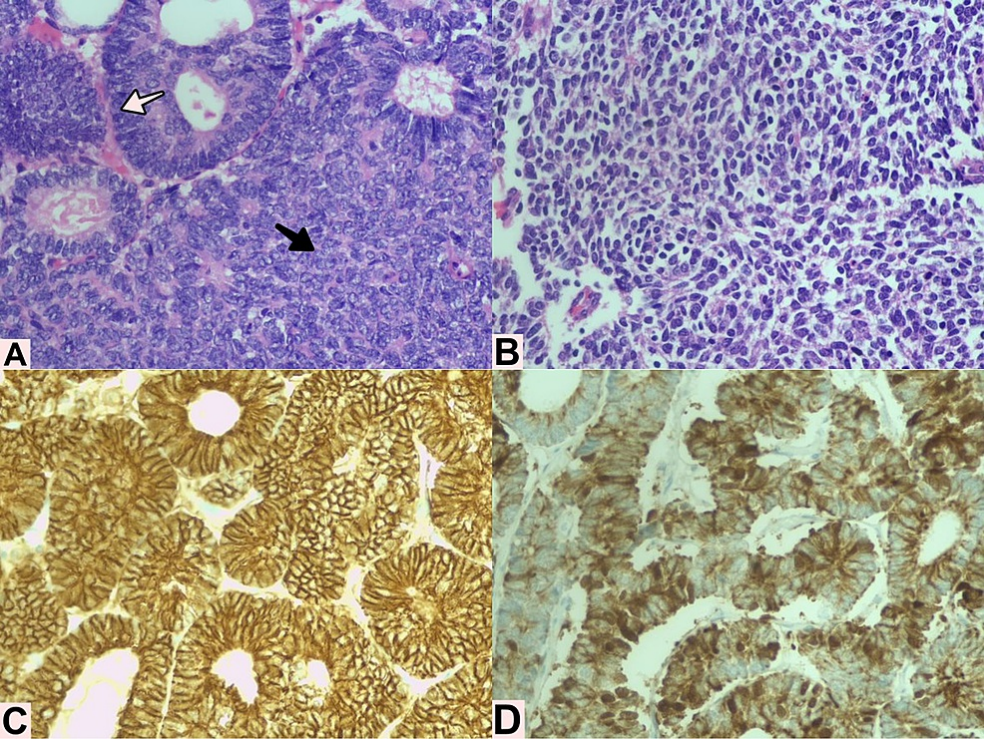
Histological findings from right hepatic lobule lesion showing a tri-phasic pattern neoplasm A. Epithelial component made of tubular structures (white arrow), and immature blastemal component (black arrow). B. Mesenchymal component of the tumor. C. Immunohistochemistry study positive for AE1-AE3. D. Immunohistochemistry study positive for BCL2.

The patient underwent right hepatectomy in February 2018, where multiple lesions with metastatic aspect were found in the right lobule and no lesions were observed in the left hepatic lobule. The pathology report confirmed the diagnostic suspicion of Wilms' tumor due to the tri-phasic pattern and the immunohistochemistry pattern (Figure 1). Unfortunately, the molecular tests for WT1, p53, FWT1, and FWT2 could not be processed due to insurance issues. She did not receive any adjuvant therapy, and due to the negative surgical borders, only observation was performed.

The patient attended follow-up with clinical oncology in August 2018, with clinical and functional decline (ECOG 2), dyspnea, cough, chest pain, and supraclavicular masses. Imaging studies were performed: in a thoracic and abdominal computed tomography (CT), pulmonary and lymph nodes progression was observed (supraclavicular, mediastinal, and retroperitoneal). The NWTS treatment scheme for stage IV was started, with a DDA4 scheme (actinomycin D, vincristine, and doxorubicine) from 29 august 2018, with overt clinical response since the second week of treatment and with no toxicity associated. No concomitant radiotherapy was offered due to the lack of evidence in adult patients and the risk of severe toxicity in this population.

The patient finalized the chemotherapy protocol in February 2019, when imaging studies found a nearly complete response, only with a non-specific right hilum lymph node of 8mm and a 4mm nodule in the left inferior lung lobule. A multidisciplinary board meeting with clinical oncologists and oncologic radiotherapists was performed, leading to the recommendation of a lung bath in a 2GY fractioning for a total dose of 12GY. The patient completed such treatment without any toxicity in May 2019. Furthermore, she underwent total abdominal radiotherapy in a 1.8GY fractioning up to a total dose of 10.8GY, completing the radiotherapy treatment in June 2019 without any toxicity.

**Figure 2:**
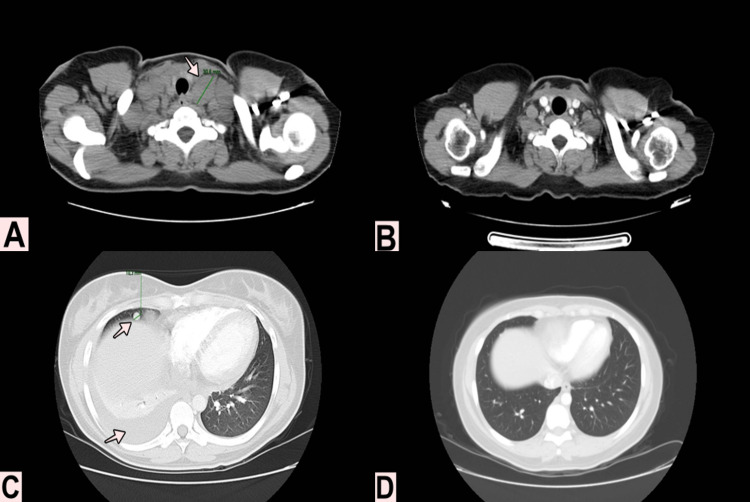
Imaging studies findings A. Neck CT from August 2018 showing a supraclavicular lymph node of 30mm. B. Neck CT from October 2019 with the resolution of supraclavicular lymph node involvement. C. Chest CT from August 2018 where right pleural effusion and pulmonary involvement were observed with a solid nodule of 10mm located in the right middle lobe. D. Chest CT from October 2019 with the resolution of the lung involvement and the pleural effusion.

In the last follow-up with clinical oncology in September 2020, the patient was asymptomatic, with ECOG 0, and imaging studies revealing complete tumoral response (Figure 2). 

## Discussion

Wilms' tumor is a renal embryonal neoplasm that occurs predominantly in the pediatric population, with around 95% of cases being diagnosed before 15 years [1-2]. An incidence of 10 cases per million persons per year in the pediatric population has been reported, in contrast to less than 0.2 cases per million persons per year in the adult population [1-3]. The typical clinical presentation in the pediatric population is an abdominal mass, painful in 50% of cases, and in less than 30% of cases, it is associated with hypertension or hematuria; bilateral renal involvement is unusual, with less than 10% of cases reported [1-4]. Clinical presentation in the adult population is similar, however, a metastatic disease within diagnosis is more common (10% in the pediatric population vs 30% in the adult population), and up to 50% of cases are in an advanced stage (III-V) [4,5]. Diagnostic delays and potential biological differences may lead to a more advanced disease at the first diagnosis in the adult population [5]. Histologically, there is no difference between adult and pediatric Wilms' tumor. WT1 gene mutation has been found in 10% of cases; 11p, 7p, 16q, and 1p changes have been found, although these are not always present. Microscopically, three components may be observed: stromal/mesenchymal, with a less aggressive behaviour, characterized by myxoid fusiform cells. The blastemal component, with the worst prognosis and more frequently found in the adult population, consisting of small cells, with scarce cytoplasm, round nuclei, and small nucleoli; finally, the epithelial component with neural line and glomerular cells [5].

The prognosis in the pediatric population has been dramatically modified in the last few decades, from a global five-year survival of 20% in 1960 to over 90% after multimodal protocols, including high-intensity chemotherapy, have been implemented [1-5]. The largest experience with Wilms' tumor in Colombia was reported in a pediatric patient cohort from Medellin. In this cohort, 84 patients with a mean age of three years were reported, predominantly stage III at the time of diagnosis and with an abdominal mass as the most frequent cardinal sign. The mean survival free of relapse was of 97 months. At 108 months after diagnosis, the survival was 71%, 20 patients presented relapse, and 11 of them died [6].

Around 300 adult Wilms' tumor cases have been reported in the literature [4]; due to the scarcity of cases, no clinical studies reporting a standard of management in the adult age are available, and in most isolated cases, the management is extrapolated from pediatric guidelines [6-13]. In the particular case of Colombia, only two adult Wilms' tumor cases have been reported, which were in an initial stage and underwent radical nephrectomy [7,8].

Two big observational studies have successfully specified clinical, therapeutic, and survival characteristics in adult patients with Wilms' tumor [14, 15]. The EUROCARE study, published in 2006, reported a cohort of adult patients with Wilms' tumor from Europe, including patients from 15 to 99 years, finding 143 patients in 16 countries, with a median age of presentation of 34 years, an annual incidence of 0.17-0.27 cases per million persons, and a global survival of 69.9% at one year after diagnosis and 47% at five years, with better survival in women than men [14]. In 2019, the biggest Wilms' tumor comparative study was published from the National Cancer Database by Satlzman et al. finding 2686 cases in children, 91 cases in young adults from 16-35 years, and 35 cases in patients older than 35 years. A global survival in five and 10 years was significantly better in children than young adults or older adults: 93.1% vs 79.1% vs 78.9% (p<0.001), and 91% vs 52% vs 70% (p<0.001), respectively [15]. The standard of treatment of pediatric Wilms' tumor usually involves multimodal therapy; even though surgery is the cornerstone of therapy, many patients will require chemotherapy or adjuvant radiotherapy to improve the probability of response and complete remission of the disease [3, 15]. In the Saltzman et al. study, a multivariate analysis with linear regression was performed, which showed how older adult patients with Wilms' tumor receive less frequent chemotherapy (OR 0.38, 95% CI 0.24 - 0.62), radiotherapy (OR 0.62 95% CI 0.4 - 0.95), or lymph node dissection at the time of surgical resection (OR 0.19, 95% CI 0.13 - 0.28) when compared to the pediatric population. This study concluded that the worse prognosis in the adult population is partially explained by modifiable factors such as inadequate stratification due to the low rate of lymph node sampling and low use of adjuvant therapies [15].

## Conclusions

Wilms' tumor has an extremely rare incidence in adults. The diagnosis is usually performed in later stages and the oncologic prognosis is considerably worse compared to children. Management based on pediatric protocols has shown improved survival outcomes. Therefore, even though no evidence of specific clinical studies performed in the adult population is available, it is reasonable to extrapolate pediatric protocols to adult patients.
